# Medical News and Miscellany

**Published:** 1897-06

**Authors:** 


### Medical News and Miscellany.

**Dr. A. A. Gregory** has located at Cleburne, Texas.

**Dr. J. A. T. Page** has removed from Wheelock to Lott.

**Dr. Virgil C. Littlefield** has located at Leesville, Texas.

**Dr. C. D. Williamson** has located at Harpersville, Texas.

**Dr. J. D. Gray** has removed from Gorman to Whitney, Texas.

**Dr. H. C. Ghent's** son has received the appointment as cadet at Annapolis.

**Dr. A. A. Blassingame** has removed from Galveston to Elmont, Texas.

**Dr. J. W. McLaughlin,** of Austin, has a son at the Johns-Hopkins studying biology.

**Dr. Ralph Steiner** has been appointed on the Board of Pension Examining Surgeons at Austin.

**The** Journal regrets to learn that Dr. A. V. Doak's son, at Taylor, Tex., met with a serious accident recently.

**Mrs. Eleanor Becton,** mother of Dr. E. P. Becton, superintendent State institute for the blind, died at the home of her daughter, Mrs. Nunnally, at Fort Worth, on the night of May 22d ult., aged 88 years.

**Married,** at Galveston, Tex., May 5th, ult., at the home of the bride's parents, Dr. Welborn Barton, of Temple, Tex., to Miss Martha E. Allen, of Galveston.

---

**Married,** at Galveston, Tex., May 18th, ult., Dr. L. E. Magnenat, of the Texas Medical College, to Miss Lucile Stone Evans, daughter of Capt. W. E. Evans, all of Galveston.

---

**Messrs. William Wood & Co.,** medical publishers, have established a State agency for Texas, at Dallas. P. O. Box No. 336, with Mr. H. R. Curtiss, of New York, as representative.

---

**Dr. Wm. A. Adams,** professor of the Principles and Practice of Medicine in the Medical Department of the University of Fort Worth, has had conferred on him the tittle of LL. D., by Mercer University.

---

**On the** 29th of May, ult., Dr. Daniel, of the TEXAS MEDICAL JOURNAL, by invitation, delivered a lecture to the law classes of the University of Texas. His subject was, "*Jurisprudence of Insanity, with especial reference to the Burt case.*"

---

**At the recent meeting** of the Brazos Valley Medical Association, at Cameron, Texas, Dr. G. M. Abney, of Franklin, was elected President, and Dr. W. B. Briggs, of Easterly, Secretary. The next meeting will be held in Navasota next November.

---

**Texas graduates** of the Memphis Hospital Medical College, March 31, 1897. Of a class of 316 students there were 65 graduates, 11 of whom were Texans. (We would give their names, but their place of residence is not published in the catalogue.)

---

**Doctors Taxed in Texas.**—One of the recent acts of the Texas Legislature, which received the Governor's signature and became a law, was an occupation tax in which doctors are to pay an annual tax of $5. Looks like reconstruction days and internal revenue.

---

**The 65th annual meeting of the British Medical Association** will be held at Montreal, August 31, and September 1, 2, and 3rd, 1887. The JOURNAL is in receipt of the preliminary programme, which is very elaborate; and also an invitation to be present is acknowledged by the editor.

**The New York Polyclinic** will have its new buildings ready in time for the fall term. This school is very popular with Texas physicians. We have something of interest to offer to a physician who may wish to attend this school during the coming fall and winter term. Address the TEXAS MEDICAL JOURNAL.

---

**The Medical Department** of Tulane University, the old Louisiana Medical College, has dispensed with the "inherited, stilted and pedantic diploma in Latin, intelligible to few others than professors and tutors of Latin," as Prof. Chaille expresses it, and has replaced it by "a brief and unpretentious diploma in English, intelligible to all." That's sense.

---

**Tulane University,** Medical Department, shows, according to report of the dean, that "at the close of its sixty-third year's existence, 3120 graduates in medicine, and 286 in pharmacy; total, 3406. The class of 1896-7 numbered 377 matriculants, and there were 100 graduates. * * * There was no valedictorian this year, owing to some misunderstanding, * * * and in lieu of the usual valedictory address by one of the graduates, Prof. Chaille gave the class, and the audience attending the closing exercises, an excellent address on "The Practice of Medicine as a Money-Making Occupation." It is most interesting and instructive reading, and we advise our younger readers to ask Dr. Chaille for a copy of it, and to study it. Great and numerous additions and improvements have been made in the college, and the facilities for teaching medicine are unsurpassed by any school in the United States.

---

**Death of Mrs. Wagley.**—The JOURNAL is in receipt of a card bearing the following inscription:

Died, in Paris, France, May 11th, 1897, Mrs. T. J. Wagley, wife of Dr. T. J. Wagley, of Cleburne, Texas. Funeral services were held at the American Church of the Holy Trinity, Avenue de l'Alma, Paris.

Mrs. Wagley had been in bad health several years. In 1891, she and Dr. Wagley spent some months in Berlin and Vienna. In Berlin was born their only child, and ever after their return to America, Mrs. Wagley felt drawn to that place, saying, too, that during their stay in Berlin her health improved; hence, to gratify her, and in the hope of benefitting her health, the doctor took her back to Europe in April, 1896. The JOURNAL extends its condolence to its esteemed friend, the bereaved husband.

**Denver is the Place.**—The medical profession of Denver are making a big effort to get the American Medical Association, which meets this month at Philadelphia, to hold its next session in the Rocky Mountain City. A large fund has been raised to entertain the delegates, and many other inducements are offered. By all means, let the West have a chance, once, at least. We vote for Denver.

---

**And,** at last, the Texas legislature, adjourned without passing any medical bill. The Association bill was gotten through the Senate; but in the House it was vigorously opposed and laid aside. It was never called up again, and hence it sleeps, as did Aaron, with its forefathers. The legislature is now in called session, but they can only legislate on subjects indicated by the governor, and he did not include medical legislation in his message. There are nine doctors in the body.

---

**Texas Graduates in Medicine and Pharmacy.**—At the annual commencement of Tulane Medical College, New Orleans, held April 17, 1897, in a class of one hundred graduates there were sixteen Texas students, fourteen graduates in medicine and two in pharmacy.

---

### Bureau of Exchange.

A good paying practice in railroad town, black land prairie, Navarro county. Good school and churches. Will induct purchases into practice. Practice given away to purchaser of residence and office. You will be pleased. Reason for selling, want to move to larger town. Write for terms, etc. Address TEXAS MEDICAL JOURNAL, Bureau of Exchange.

